# Aspirin Resistance and Promoting Blood Circulation and Removing Blood Stasis: Current Situation and Prospectives

**DOI:** 10.1155/2014/954863

**Published:** 2014-02-18

**Authors:** Jie Wang, Xingjiang Xiong, Bo Feng

**Affiliations:** Department of Cardiology, Guang'anmen Hospital, China Academy of Chinese Medical Sciences, Beixiange 5, Xicheng District, Beijing 100053, China

## Abstract

Aspirin plays a crucial physiological and pathophysiological role in cardiovascular diseases and cerebrovascular diseases by irreversibly inhibiting thromboxane A2. However, some patients may be “resistant” to its effect. The resistance has close association with adverse cardiovascular outcomes and increased mortality, so that resolving the problem of aspirin resistance (AR) is widely concerned. By studying the correlation between AR and blood stasis syndrome (BSS), it is demonstrated that BSS may be one of the pathogenesis of AR in traditional Chinese medicine. Chinese herb and formulas definitely possess the advantage of whole body regulation by many ways and many targets. It is a new direction for treatment of AR to combine TCM and modern medicine to study the mechanism and prevention of AR.

## 1. Introduction 

**Figure 1:**
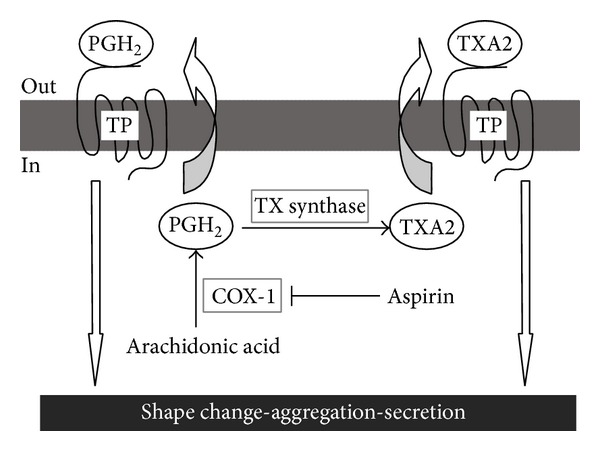
Aspirin inhibits COX-1 dependent TXA2 production.

Despite recent medical advances, cardiovascular diseases (CVDs) remain the primary cause of morbidity and mortality throughout the world [1]. Activation of platelets plays a crucial physiological and pathophysiological role in thromboembolic events such as myocardial infarction, stroke, and acute limb ischemia [2]. Hence, drugs that inhibit platelet aggregation, particularly aspirin, are of essential significance for cardiovascular prevention. Currently, evidence of a number of large-scale clinical trials demonstrated that aspirin is a cornerstone in the primary and secondary prevention of CVDs [3, 4]. It is widely used in the medical management of acute coronary syndromes, in the prophylaxis of patients undergoing percutaneous angioplasty or vascular grafting and in long-term prevention of cardiovascular and cerebrovascular events [5]. Meta-analysis of 197 randomized controlled trials involving 135640 patients demonstrated that aspirin reduces the risk of a serious vascular event or cardiovascular death by 25% in high-risk patients [6]. Clinical and biochemical evidence revealed that a persistent thromboxane A2 (TXA2) production is the most likely cause of the residual platelet function [7]. Aspirin covalently and irreversibly inhibits COX-1 and COX-2 by acetylation of serine 530 and serine 516 in their respective active sites. Recent study showed that aspirin inhibits COX-1 approximately 170-fold more potently than COX-2 [8]. In short, aspirin can mainly affect the TXA2 pathway which is the single pathway of platelet activation by irreversibly inhibiting cyclo-oxygenase-1 (COX-1) by acetylation of serine 530, induce long lasting functional defects of the platelets, and display good antithrombotic activity [9, 10] (as shown in Figure 1).

However, some patients may be “resistant” to its effect. AR is widely manifested and associated with increased cardiovascular risk in aspirin-treated patients. This side effect can significantly influence the clinical effect. Measuring response to aspirin is often difficult and there is no accepted definition of AR [11]. On current, several definitions for resistance have been set. Some scholars defined “aspirin resistance” as the inability of aspirin to inhibit COX-1 dependent TXA2 production and consequently TXA2-dependent platelet functions [12]. Arguably, the most fundamental definition of AR is lower than the normal antiplatelet response to standard doses of aspirin [13]. That is to say, the term AR may mean different things to different individuals [7]. Studies in adults reported a 5%–51% prevalence of aspirin resistance, while the ratio was 26% in children [14]. AR may be associated with an increased risk of dying from heart disease [15]. Nowadays, a prevalence investigation in Indian showed that incidence of AR in the cohort of patients with documented heart disease was 38.1% [16]. Several laboratory methods have been proposed to assess platelets' resistance (bleeding time, light transmission aggregation, impedance aggregation, platelet function analyser, rapid platelet function assay, TXB2, and flow cytometry), of which light transmittance aggregometry (LTA) is considered gold standard [17]. Besides there are also a variety of other techniques to assess aspirin responsiveness and there are advantages and drawbacks with each.

There are several proposed mechanisms of aspirin resistance, and true aspirin resistance is unclear. Aspirin noncompliance, insufficient dosing, platelet turnover, and interacting drugs, most notably nonsteroidal inflammatory drugs (NSAIDs), unfortunately are the most important contributors to the treatment failure. Aspirin resistance is a multifactorial phenomenon, so strategies of treatment should be directed to a number of COX-1 dependent and other independent factors, such as increasing patient compliance, increasing dose of levels, combining with other antiplatelet drugs, or applicating other kinds of drugs as alternatives. Also, adequate treatment of confounding clinical conditions such as smoking, hyperlipidemia, hyperglycemia, hypertension, heart failure, infection, and inflammation may further increase efficacy of antiplatelet treatment with aspirin (Table 1). However, there also exists similar phenomenon to AR, such as clopidogrel resistance (CR). On the other hand, the combination of the two types of antiplatelet drugs may cause increasing risk of serious bleeding. Considering the higher price of clopidogrel and other drugs, it is not suitable for patients for long-term use [18]. That is to say, it is not an ideal therapy to combine aspirin with other antiplatelet drugs or alternative medication. In such cases, we can seek solution from traditional Chinese medicine (TCM).

## 2. **The Pathogenesis of AR in TCM Theory**

With increasing application of complementary and alternative medicines all over the world, TCM became more popular and have drew more attention [19–23]. It has formed a particular way on diagnosis and treatment of disease [24–28]. Currently, blood stasis syndrome (BSS) and theory of promoting blood circulation and removing blood stasis (PBCRBS) is attached by scholars from various countries. In America, doctors are familiar to ABC drugs (activating blood circulation herbs), which were herbs with function of PBCRBS [29–32]. In Japan, doctors call BSS as Oketsu Syndrome [33–35]. In TCM, cardiovascular heart disease (CHD) belongs to category of “thoratic obstruction and cardialgia” or “chest stuffiness and pains.” Ancient Chinese medicine had some understanding about thromboembolic disease. The “Five Pathogenic Factors” in the ancient book of “the Miraculous Pivot” recorded that “pathogenic factor (also called “xie qi”)” lies in heart, so the disease is manifested as precordial pain.” The pathogenesis of AR is related to obstruction of collaterals by blood stasis and obstruction of heart meridian. By theory of TCM, AR has a close association with cardiovascular and cerebrovascular thromboembolic diseases, especially the cardiovascular heart disease. BSS is the main syndrome of CHD and it is an important TCM syndrome of CHD with cardiovascular risk [36]. BSS is defined as clinical syndrome caused by retardation or cessation of the blood flow and is regarded as the cause or product of many chronic diseases in TCM [37]. Recognition of ancient Chinese medicine to blood stasis is multifaceted and there were many narration about it. For example, there were 365 kinds of Chinese herbal medicine in “Classic of Materia Medica by God of Agriculture” (Shennong Ben Cao Jing), of which 41 kinds have function of PBCRBS. At least the following diseases of cardiovascular system may be related to manifestation of BSS, such as angina pectoris, acute myocardial infarction, rheumatic heart disease, heart failure, and various types of vasculitis [38, 39].

By theory of TCM, AR belongs to the category of “collaterals” disease. The pathological character of AR is blood stagnant. Accumulation of blood stasis can be transformed into turbid toxins. Finally, the blood stasis and the turbid toxin congest the “collaterals” and leaded to various diseases. In view of the characteristics of the disease, strategies of PBCRBS and removing toxic materials from meridians should be used to AR. AR will eventually lead to formation of BSS and cause blood stasis in channels and collateral branches by all kinds of reasons. Orient scholars studied the association of AR and BSS and concluded that AR is closely related to BSS [40–42]. BSS maybe pathogenesis of AR in TCM. Firstly, as we know, the clinical manifestation of platelet function disorder is one of indicators comprising diagnosis of BSS; also this indicator is a significant factor causing AR. Also, it is indicated that the incidence rate of AR was up to 64% in patients of cardiocerebral artery thrombotic disease with severe blood stasis, which is significantly more than non-BSS patients. Secondly, the pathogenesis of BSS is correlated with haemorheologic changes such as the deterioration of erythrocyte deformability, elevation of blood viscosity, and acceleration of erythrocyte aggregation, as well as abnormal status of microcirculatory dysfunction [43, 44], which is similar to mechanism of AR. Thirdly, Chinese herbs for treatment of BSS can also improve AR [45]. Study about mechanism of traditional Chinese herbs with function of PBSRBS showed that it can not only activate blood circulation (improving function of cardiovascular and cerebrovascular, changing the physical and chemical properties of blood, changing function of platelet and blood coagulation system, and improving microcirculation) but also remove stasis (antimyocardial ischemia, anticerebral ischemia, inhibiting platelet aggregation, anticoagulation, antiformation of thrombosis, etc.). In view of this, Chinese herbs for the therapy of BSS can show significant effect on AR aiming at the pathogenic mechanism.

## 3. Principles of AR Treatment in Traditional Chinese Medicine

Dysfunction of “zang” and “fu” can lead to blood stasis of heart meridian. So that therapeutic of PBCRBS is the first choice of CHD. But, in concrete applications, it is imperative to differentiate TCM syndrome according to the etiology and pathogenesis, the involved zang-fu organs, and clinical manifestation of BSS when using PBCRBS as the main therapy. Also, it is necessary to modulate the liver, the spleen, the lung, and the kidney at the same time and use the corresponding treatment to improve effect of diagnosis and treatment, such as promoting qi circulation, dispelling cold, resolving phlegm, clearing away heat, supplementing qi, supplementing blood, warming yang, nourishing yin, and supplementing kidney. Chinese herbs of PBCRBS have effect on antibacterial, antiviral, inhibiting inflammation, treatmenting infectious diseases, regulating immune system, improving body resistance, and so on [46]. Besides, recent research demonstrated that CHD is closely related to inflammation, so that maybe principal of dissolving blood stasis and detoxification for treatment of BSS is more effective. Current researches demonstrated that Chinese herb and formulas definitely possess the advantage of whole body regulation by many ways and many targets, so that we can resolve AR issues with higher efficacy to prevent the occurrence of cardiovascular disease.

Nowadays, alternative and synergistic treatment of TCM has become an ideal therapeutics. Researches demonstrated that some Chinese herbal compounds (such as composite salvia dropping pill, Nao xin tong capsule, Tong xin luo capsule, Xuefu zhuyu decoction, Huo xue capsule, and Zhilong huoxue capsule) and several traditional medicine monomer or Chinese herbal extract (Di'ao xinxuekang capsule, Xinnao shutong capsule, ginkgo biloba extract, etc.) can effectively inhibit platelet activity. These drugs can be used combining with aspirin to achieve the purpose of enhancing pharmacy efficiency. Also, it can be used as alternative medicine of aspirin for secondary prevention of cardiovascular and cerebrovascular diseases [47]. It is demonstrated that most of the decoctions have effect on promoting blood circulation to remove stasis and this fact can also verify the previous mentioned theory.

## 4. TCM of PBCRBS for the Treatment of AR

Currently, traditional medicine monomer and Chinese formulas used alone or combined with antiplatelet pertensive drugs have been widely used as an alternative and effective method for the treatment of AR with coronary heart diseases in clinical treatment in China. Until now, many clinical studies of formulas used for therapy of AR verified the clinical effect ranging from case reports and case series to controlled observational studies and randomized clinical trials. However, there is no critically appraised evidence such as systematic reviews or meta-analyses on potential benefits and harms of these Chinese formulas for AR to justify their clinical use and their recommendation. This paper aims to assess the current clinical evidence of the frequently used Chinese decoctions or traditional medicine monomer for aspirin resistance in patients with coronary heart diseases.

We selected all the clinical trials about Chinese decoctions or traditional medicine monomer for treatment of AR using the search terms of “aspirin resistance,” “coronary heart disease,” “herb,” “Chinese drug,” “compound prescription,” “traditional Chinese medicine,” “decoction,” “Chinese formula,” “controlled clinical trial,” “random,” and “blind” individually or combined in five databases, such as Chinese National Knowledge Infrastructure (CNKI), Chinese Biomedical Literature Database (CBM), PubMed, Embase, and the Cochrane Central Register of Controlled Trials in the Cochrane Library (January 2013). After primary search of five databases, ten RCTs were reviewed [48–57]. All the trials were conducted in China and published in Chinese. The characteristics of the fourteen randomized trials were summarized in Table 2. The majority of the included trials were assessed to be of general poor methodological quality according to the predefined quality assessment criteria. The randomized allocation of participants was mentioned in all trials. However, only 2 trials stated the methods for sequence generation by random number table [51, 56].

751 patients with AR were included. Intervention included all the prescriptions of Chinese decoctions or traditional medicine monomer, or combined with antiplatelet-aggregation drugs. The Chinese decoction includes composite salvia dropping pill, Huo xue capsule, Nao xin tong capsule, Tong xinluo capsule, Xuefu zhuyu decoction, and Zhilong huoxue capsule. The traditional medicine extract or monomer can only be reported by five researches [53, 56, 57], such as Di'ao xinxuekang capsule, Ginkgo biloba extract, and Xinnao shutong capsule. The different ingredient of frequently used Chinese formulas is shown in Table 3. The controls included antiplatelet-aggregation drugs alone. The standard therapy of control group included aspirin (100 mg, qd) [48–51, 53, 54, 57], routine treatment plus clopidogrel (75 mg, qd) [52], and aspirin (300 mg, qd) [55, 56]. Only three trials investigated the prescriptions of Chinese medicine used alone versus antiplatelet-aggregation drugs [52, 55, 56]. The other eleven clinical trials investigated the Chinese medicine combined with antiplatelet-aggregation drugs as therapy intervention versus antiplatelet-aggregation drugs [49–51, 53, 54, 57].

The treatment duration ranged from 14 days to 1 year. All of the 10 trials used platelet aggregation as the outcome measure. In addition, the outcome measurement also includes the unfrequently used standards, such as uric acid, blood lipid, CRP, hs-CRP, TXB2, COL, 6-K-PGF1*α*, platelet counts, and coagulation time. The main finding showed that the combining use of Chinese medicine and antiplatelet-aggregation drugs can decrease the platelet aggregation rate in a different degree more effectively. Of the ten trials, only one research does not show specific data [50].

The meta-analysis showed significant beneficial effect of Chinese formula plus antiplatelet-aggregation drugs compared with antiplatelet-aggregation drugs in platelet aggregation rate induced by AA (MD: −9.36 [−10.48, − 8.25]; *P* < 0.00001) and ADP (MD: −17.61 [−19.56, − 15.65]; *P* < 0.00001). Also, there was beneficial effect of Chinese formula used alone compared with antiplatelet-aggregation drugs in platelet aggregation rate induced by AA (MD: −2.53 [−4.28, − 0.78]; *P* = 0.005). However, the meta-analysis showed no significant effect of Chinese formula used alone compared with antiplatelet-aggregation drugs in platelet aggregation rate induced by ADP (MD: 0.70 [−11.07, 12.48]; *P* = 0.91) (as shown in Tables 4 and 5).

Six out of ten trials mentioned the adverse effect [50, 51, 53, 55–57]. Two trials reported six specific symptoms in Chinese formulas including distending feeling in head, dizziness, headache, facial flush, abdominal discomfort, and nausea. And adverse events were found in both of two trials [55, 56]. Three trials reported adverse effect in aspirin group. Two of it showed symptoms including distending feeling in head, dizziness, headache, facial flush, abdominal discomfort, and nausea [55, 56]. Another one trial reported symptoms of faulty in the mouth, gastrointestinal discomfort, gum haemorrhage, retinal hemorrhage, and positive of fecal occult blood test [53].

Our previous meta-analysis demonstrated that Chinese decoctions or traditional medicine monomer for treatment of AR showed significant beneficial effect compared with Western medicine. Adverse effect of Chinese formulas was reported by Luo et al. and Xiu [55, 56]. The adverse effect was not severe, and it spontaneously recovered without special treatment. Five trials demonstrated that there was no significant difference between the two treatments. Six trials did not mention adverse effect. Therefore, due to the limited and inadequate evidence provided by the eligible trials, conclusions about the safety of Chinese formulas combined with antiplatelet-aggregation drugs cannot be made from this paper. Large-scale clinical trials with long-term follow-up were warranted to assess the safety of new integrative medicine therapy properly.

## 5. Conclusion and Prospective

BSS is defined by retardation or cessation of the blood flow and is regarded as the cause or product of many chronic diseases. It is a diagnosis that indicates a very strong sense of TCM [58–61]. Usually, chronic diseases and slow progressing diseases mostly involve blood stasis. Also, severe warm diseases and trauma mostly involve acute blood stasis. Ancient Chinese medical texts describe commonly phenomenon of a disorder in the blood circulation as “Yu Xue” in Chinese, “Eohyul” in Korean, and “Oketsu” in Japanese [62, 63]. It has close correlation with various diseases. In cardiovascular system, there was close association between BSS and coronary artery disease, angina pectoris, myocardial infarction, rheumatic heart disease, heart failure, and vasculitis. With increasing popularity of complementary and alternative medicine among AR patients, TCM is becoming more and more frequently used both in China and Western countries [64–66]. Clinical use of PBCRBS herbs in associated diseases also becomes more plausible. Their therapeutic effect can be clearly shown. Thus, the “PBCRBS phenomenon” becomes more widely discussed and becomes a hot topic of integrative medicine.

Statistical analysis on sixteen Chinese traditional medicine materia medica classics showed that there are 150 commonly used PBCRBS herbs. In addition, Chinese herbal medicine can be widely used for AR. We statis frequency of each Chinese herbs used for aspirin resistance in current frequently Chinese formulas; the frequent appearance of herbs is hirudo. Hirudo, also called leech, the herb is some bitter and salty in flavor and enters liver and bladder meridian. Modern pharmacological studies demonstrated that the herb contains peptides, heparin, and antithrombin [67]. Besides, at least four experiments confirmed that leech and its effective ingredients can reduce or inhibit activation of platelet [68–71]. Furthermore, the semen persicae, radix paeoniae rubra, and radix astragali were used frequently, of which, semen persicae is a commonly used traditional Chinese herbal medicine with function of PBCRBS. It showed therapeutic effect on treatment of coronary heart disease, myocardial infarction, and traumatic injury. Related study showed that semen persicae can effectively inhibit platelet aggregation by multiple pathways [72]. Radix paeoniae rubra is another commonly used traditional Chinese herbal medicine with function of PBCRBS. It is some bitter and slightly cold in flavor and enters liver meridian and has function of resolving blood stasis, relieving pain, cooling the blood and detumescence. The paeonilorin demonstrated the effective pharmacological components (such as monoterpene and glycosides), gallic acid and its derivatives. Total paeony glycoside (TPG) is one of the main effective components of radix paeoniae rubra. It has been demonstrated that TPG has anticoagulant and antithrombotic effects [73]. The animal experiment showed that TPG can improve the microcirculation of mice, reduce serum and plasma viscosity in rats, and inhibit platelet aggregation induced by adenosine diphosphate (ADP) [74]. Radix astragali was mostly used for qi deficiency disease. According to theory of traditional Chinese medicine, invigorating qi can promote blood circulation. So radix astragali can be used for treatment of BSS combined with PBS herbs. Bu yang huan wu decoction is a typical example using this treatmen rule [75]. At present, at least five experiments showed that astragalus could reduce platelet adhesion and aggregation, reduce plasma fibrinogen, and show antithrombus formation effect [76–79]. In addition, Panax notoginseng, salvia miltiorrhiza, and bidentate achyranthes were also used frequently.

Under what circumstance can we use PBCRBS? For a long time, PBCRBS herbs were maily used for traumatic injury, “zhengjia” agglomeration, and gynecological diseases. They were seldom used in treatment of coronary heart disease. Professor Chen keji sticks to the point that we should associate the main pathological link of the coronary disease (such as formation of thrombosis, platelet activation, vascular stenosis, and spasm) with BSS, so that etiology and pathogenesis of the disease can be clearly clarified by theory of TCM. Traditionally, the diagnosis of BSS depended on subjective diagnostic methods such as inspection and palpation of the patient [80]. In 1988, academia from Japan, Korea, Singapore and so forth attended the “International Conference on Blood Stasis Syndrome” and recognized a standard for diagnosing blood stasis syndrome. In China, Chen improved the above standards and grading system in more detail and formed a commonly used Chinese criteria diagnosis of BSS [81].

There are still some problems existing which seriously limited the research and progress on the treatment and should be solved as soon as possible. Researchers of clinical trials in TCM should also pay more attention to experimental design and methodological quality and improve the reporting quality according to the consolidated standards of reporting trials (CONSORT) [82]. Also, it is imperative to establish a rapid, accurate, and practical method for monitoring of AR. Monitoring platelet activity can be just as easy as blood pressure, blood glucose, and cholesterol. Currently, Chinese scholars study the mechanism of BSS mainly on direction of microcirculation, hemorheology, platelet function, deformability of red blood cell, prostaglandin, thromboxane metabolism, and fibrinolytic system change. There were many researches duplicating previous work. So that, it is necessary to carry out further targeted research and design observation index according to different kinds of diseases. In addition, Chinese scholars used too many types of decoctions for treatment of BSS. Maybe it is better to concentrate on study of the generally accepted formulas or decoctions. In addition, the abuse in using Chinese herbs for treatment BSS should be avoided. Caution should a1ways be taken to differentiate clinical application.

## Figures and Tables

**Table 1:** Causes of aspirin resistance and potential strategies to overcome resistance.

Cause of AR	Strategy to overcome AR
Noncompliance of patients	Patient education Minimisation of adverse effects
Insufficient dosing	Biofeedback use of platelet function assays and measurement of aspirin metabolites to guide increased dosing levels
Drug-drug interactions	Caution/avoid use of NSAIDs Caution/avoid use of proton pump inhibitors
Non-COX-1-mediated TXA2 production	Duel or increased dose of antiplatelet therapy Development of thromboxane receptor antagonists
Other pathways of platelet activation	Use of drugs to inhibit final common pathways of platelet aggregation
Pharmacogenetic factors	Exploitation of genetic polymorphisms-personalised antiplatelet therapy

**Table 2:** Clinical trials of Chinese herbal interventions in treating AR with a concomitant population.

Reference (year)	Study design	Participants T/C	Intervention (herb included)	Control	Outcome measure	Treatment duration	Main finding (*P* value)
Chai et al. (2008) [48]	RCT	10/10	Composite salvia dropping pill (10 pills, tid); aspirin (100 mg, qd)	Aspirin (100 mg, qd)	Platelet aggregation rate induced by AA and ADP	2 w	Composite salvia dropping pill can enhance patients' sensitivity to aspirin. The combining use can synergisticly exert antiplatelet effect.

Peng et al. (2011) [49]	RCT	12/13	Huo xue capsule (4 pills, tid); aspirin (100 mg, qd)	Aspirin (100 mg, qd)	Platelet aggregation rate induced by AA and ADP	12 w	The combination of huo xue capsule and aspirin can decrease the platelet aggregation rate significantly.

Yang et al. (2011) [50]	RCT	130/130	Nao xin tong capsule (2 pills, tid); aspirin (100 mg, qd)	Aspirin (100 mg, qd)	Platelet aggregation rate induced by AA and ADP, the incidence of AR and ASR of each group.	24 w	The combining use of nao xin tong capsule and aspirin can significantly decrease the platelet aggregation rate. Incidence of AR and ASR is lower than single aspirin group.

Yin et al. (2010) [51]	RCT	30/29	Tong xin luo capsule (3 pills, tid); aspirin (100 mg, qd)	Aspirin (100 mg, qd)	COL and platelet aggregation rate induced by AA and ADP	30 d	Tong xin luo capsule has some effects on decreasing the platelet aggregation rate induced by ADP and COL.

Song (2008) [52]	RCT	24/23	Tong xinluo capsule (4 pills, tid)	Clopidogrel (75 mg, qd)	CRP, TXB2, and platelet aggregation rate induced by AA and ADP	30 d	Tongxinluo capsule has antiplatelet aggregation and anti-inflammatory effect in ACS patients with AR and can improve AR in ACS patients in a certain degree.

Liu (2011) [53]	RCT	30/30	Xinnao shutong capsule (9 pills, qd); aspirin (100 mg, qd)	Aspirin (100 mg, qd)	Uric acid, Blood lipid, Hs-CRP, TXB2, and platelet aggregation rate induced by AA and ADP AA, ADP, and so on.	1 y	The combining use of aspirin and xinnao shutong capsule can decrease the adverse cadiovascular events of patients with AR.

Wu (2012) [54]	RCT	30/30	Xuefu zhuyu decoction (1 dose, qd); aspirin (100 mg, qd)	Aspirin (100 mg, qd)	The expression of AA and ADP in serum.	4 w	The combining use of aspirin and Xuefu zhuyu decoction can significantly decrease the platelet aggregation rate induced by ADP and AA.

Luo et al. (2012) [55]	RCT	30/30	Zhilong huoxue capsule (1.6 g, tid)	Aspirin (300 mg, qd)	The mean platelet aggregation rate, TXB2, and 6-K-PGF1*α*.	4 w	Capsule zhilong huoxue of aspirin resistance has better efficacy and safety. The mechanism may be related to its effect of decreasing TXB2, increasing 6-K-PGF1*α*, and decreasing TXB2/6-K-PGF1*α*.

Xiu (2012) [56]	RCT	30/30	Di'ao xinxuekang capsule (1.6 g, tid)	Aspirin (300 mg, qd)	Platelet aggregation rate induced by AA and ADP	4 w	Capsule di'ao xinxuekang of aspirin resistance has better efficacy and safety. The mechanism may be related to its effect of decreasing TXB2, increasing 6-K-PGF1*α*, and decreasing TXB2/6-K-PGF1*α*.

Yang et al. (2011) [57]	RCT	50/50	Ginkgo biloba extract (19.2 mg, tid); aspirin (100 mg, qd)	Aspirin (100 mg, qd)	Platelet aggregation rate induced by AA, ADP, and FAP	2 w	Combination of ginkgo biloba extract and low dosage of aspirin can decrease AR and decrease incidence of adverse cadiovascular events.

Notes: T/C: treatment group and control group; CT: clinical trial; RCT: randomized clinical trial; NR: not reported; ADP: adenosine diphosphate; COL: collagen.

**Table 3:** The ingredient of frequently used Chinese Formulas.

Formulas	Components	TCM Efficacy
Composite salvia dropping pill	Salvia miltiorrhiza [Dan shen, *丹参*], Panax notoginseng [San qi, *三七*], and Borneolum Syntheticum [Bing pian, *冰片*].	Promoting blood circulation to remove stasis and regulating the circulation of qi and alleviating pain.

Huo xue capsule	Radix astragali [Huang qi, *黄芪*], Semen persicae [Tao ren, *桃仁*], Safflower [Hong hua, *红花*], Bidentate achyranthes [Niu xi, *牛膝*], *酸枣仁*, Rhizoma chuanxiong [Chuan xiong, *川芎*], Citrus aurtantium [Zhi qiao, *枳壳*], Radix rehmanniae [Di huang, *地黄*], Platycodon grandiflorum [Jie geng, *桔梗*], Angelica sinensis [Dang gui, *当归*], and Glycyrrhiza [Gan cao, *甘草*].	Suplementing qi, nourishing blood, promoting blood circulation for removing obstruction, and regulating qi-flowing for tranquilization.

Nao xin tong capsule	Radix astragali [Huang qi, *黄芪*], Radix Paeoniae Rubra [Chi shao, *赤芍*], Salvia miltiorrhiza [Dan shen, *丹参*], Angelica sinensis [Dang gui, *当归*], Rhizoma chuanxiong [Chuan xiong, *川芎*], Semen persicae [Tao ren, *桃仁*], Olibanum [Ru xiang, *乳香*], Myrrha [Mo yao, *没药*], Bidentate achyranthes [Niu xi, *牛膝*], Ramulus cinnamomi [Gui zhi, *桂枝*], Ramulus mori [Sang zhi, *桑枝*], Earthworm [Di long, *地龙*], Caulis Spatholobi [Ji xue teng, *鸡血藤*], Scorpio [Quan xie, *全蝎*], and Hirudo [Shui zhi, *水蛭*].	Benefiting qi for activating blood circulation and promoting blood circulation for removing obstruction in collaterals.

Tong xin luo capsule	Radix Ginseng [Ren shen, *人参*], Hirudo [Shui zhi, *水蛭*], Scorpio [Quan xie, *全蝎*], Eupolyphaga Seu Steleophaga [Tu bie chong, *土鳖虫*], Scolopendra [Wu gong, *蜈蚣*], Periostracum Cicadae [Chuan tui, *蝉蜕*], Radix Paeoniae Rubra [Chi shao, *赤芍*], Lignum Santali Albi [Tan xiang, *檀香*], Lignum Dalbergiae Odoriferae [Jiang xiang, *降香*], Olibanum [Ru xiang, *乳香*], Semen Ziziphi Spinosae [Suan zao ren, *酸枣仁*], and Borneolum Syntheticum [Bing pian, *冰片*].	Benefiting qi for activating blood circulation and promoting blood circulation for removing obstruction in collaterals and relieving pain.

Zhilong huoxue tongyu capsule	Radix astragali [Huang qi, *黄芪*], Radix Ginseng [Ren shen, *人参*], Hirudo [Shui zhi, *水蛭*], Ramulus cinnamomi [Gui zhi, *桂枝*], Caulis Spatholobi [Ji xue teng, *鸡血藤*], Earthworm [Di long, *地龙*], and others.	Benefiting qi for activating blood circulation and removing obstruction in collaterals.

Xuefu zhuyu decoction	Semen persicae [Tao ren, *桃仁*], Angelica sinensis [Dang gui, *当归*], Citrus aurtantium [Zhi qiao, *枳壳*], Rhizoma chuanxiong [Chuan xiong, *川芎*], Bupleurum [Chai hu, *柴胡*], Safflower [Hong hua, *红花*], Bidentate achyranthes [Niu xi, *牛膝*], Radix Paeoniae Rubra [Chi shao, *赤芍*], Radix rehmanniae [Di huang, *地黄*], Platycodon grandiflorum [Jie geng, *桔梗*], and Glycyrrhiza [Gan cao, *甘草*].	Promoting blood circulation to remove stasis and regulating the circulation of qi and alleviating pain.

Di'ao xinxuekang capsule	Extract from root of haicaet Burkill [Huang shan yao, *黄山药*] and Dioscorea niponica Makino [Chuan long shu yu, *穿龙薯蓣*]	Promoting blood circulation for removing obstruction and stimulating qi circulation to relieve pain.

Xinnao shutong capsule	Extract from aboveground part of Sandbur [Ji li, *蒺藜*]	Resolving stagnation for alleviation of pain and activating blood circulation to dredge channel blockage.

Ginkgo leaf extract	Extract from leaf of Ginkgo [Yin xing, *银杏*]	Benefiting qi for activating blood circulation and removing obstruction in collaterals.

**Table 4:** Analysis of platelet aggregation rate induced by AA.

Trials		MD (95% CI)	*P* value
Chinese formula versus anti-platelet-aggregation drugs			
Tong xin luo capsule versus clopidogrel	1	0.86 [−3.38, 5.10]	0.69
Zhilong huoxue capsule versus aspirin	1	−3.23 [−7.34, 0.88]	0.02
Di'ao xinxuekang capsule versus aspirin	1	−3.23 [−7.34, 0.88]	0.02

Meta-analysis	3	−2.53 [−4.28, −0.78]	0.005

Chinese formula plus anti-platelet-aggregation drugs versus anti-platelet-aggregation drugs			
Composite salvia dropping pill plus aspirin versus aspirin	1	−11.09 [−14.84, −7.34]	<0.00001
Huo xue capsule plus aspirin versus aspirin	1	−14.22 [−16.59, −11.85]	<0.00001
Xinnao shutong capsule plus aspirin versus aspirin	1	−12.58 [−14.56, −10.60]	<0.00001
Xuefu zhuyu decoction plus aspirin versus aspirin	1	−7.20 [−11.73, −2.67]	0.002
Ginkgo biloba extract plus aspirin versus aspirin	1	−2.53 [−4.53, −0.53]	0.01

Meta-analysis	5	−9.36 [−10.48, −8.25]	<0.00001

**Table 5:** Analysis of platelet aggregation rate induced by ADP.

Trials		MD (95% CI)	*P* value
Chinese formula versus anti-platelet-aggregation drugs			
Tong xin luo capsule versus clopidogrel	1	13.32 [7.68, 18.96]	<0.00001
Zhilong huoxue capsule versus aspirin	1	−5.50 [−10.49, −0.51]	0.03
Di'ao xinxuekang capsule versus aspirin	1	−5.50 [−10.49, −0.51]	0.03

Meta-analysis	3	0.70 [−11.07, 12.48]	0.91

Chinese formula plus anti-platelet-aggregation drugs versus anti-platelet-aggregation drugs			
Composite salvia dropping pill plus aspirin versus aspirin	1	−13.92 [−19.58, −8.26]	<0.00001
Huo xue capsule plus aspirin versus aspirin	1	−40.97 [−49.64, −32.30]	<0.00001
Tong xin luo capsule plus aspirin versus aspirin	1	−2.51 [−4.46, −0.56]	0.01
Xinnao shutong capsule plus aspirin versus aspirin	1	−23.26 [−26.11, −20.41]	<0.00001
Xuefu zhuyu decoction plus aspirin versus aspirin	1	−16.82 [−22.12, −11.52]	<0.00001
Ginkgo biloba extract plus aspirin versus aspirin	1	−2.87 [−7.00, 1.26]	0.17

Meta-analysis	5	−17.61 [−19.56, −15.65]	<0.00001

## Abbreviations

PBCRBS: Promoting blood circulation and removing blood stasis
AR: Aspirin resistance
BSS: Blood stasis syndrome
TCM: Traditional Chinese medicine
CVDs: Cardiovascular disease
TXA2: Thromboxane A2
COX-1: Cyclo-oxygenase-1
LTA: Light transmittance aggregometry
CR: Clopidogrel resistance
CHD: Coronary heart diseases
TPG: Total paeony glycoside
ADP: Adenosine diphosphate.
